# Molecular Characterization and Development of Real-Time PCR Assay for Pine-Wood Nematode *Bursaphelenchus xylophilus* (Nematoda: Parasitaphelenchidae)

**DOI:** 10.1371/journal.pone.0078804

**Published:** 2013-11-11

**Authors:** Weimin Ye, Robin M. Giblin-Davis

**Affiliations:** 1 Nematode Assay Section, Agronomic Division, North Carolina Department of Agriculture & Consumer Services, Raleigh, North Carolina, United States of America; 2 Fort Lauderdale Research and Education Center, University of Florida/IFAS, Davie, Florida, United States of America; Naval Research Laboratory, United States of America

## Abstract

*Bursaphelenchus xylophilus*, the pine-wood nematode (PWN), is the causal agent of pine wilt disease, one of the most damaging emerging pest problems to forests around the world. It is native to North America where it causes relatively minor damage to native conifers but is labeled an EPPO-A-2 pest and a quarantine nematode for many countries outside of the United States because of its potential for destruction to their native conifers. Exports of wood logs and commodities involving softwood packaging materials now require a lab test for the presence/absence of this regulated nematode species. We characterized the DNA sequences on the ribosomal DNA small subunit, large subunit D2/D3, internal transcribed spacer (ITS) and mitochondrial DNA cytochrome oxidase subunit one on the aphelenchid species and described the development of a real-time-PCR method for rapid and accurate identification of PWN targeting the ITS-1. A total of 97 nematode populations were used to evaluate the specificity and sensitivity of this assay, including 45 populations of *B. xylophilus* and 36 populations of 21 other species of *Bursaphelenchus* which belong to the *abietinus*, *cocophilus*, *eggersi*, *fungivorus*, *hofmanni*, *kevini*, *leoni*, *sexdentati*, and *xylophilus* groups and one unassigned group from a total of 13 groups in the genus *Bursaphelenchus*; 15 populations of *Aphelenchoides besseyi*, *A. fragariae*, *Aphelenchoides* species and *Aphelenchus avenae*; and one population of mixed nematode species from a soil sample. This assay proved to be specific to *B. xylophilus* only and was sensitive to a single nematode specimen regardless of the life stages present. This approach provides rapid species identification necessary to comply with the zero-tolerance export regulations.

## Introduction

The pine-wood nematode (PWN), *Bursaphelenchus xylophilus* (Steiner & Buhrer, 1934) Nickle, 1970 ( = *B. lignicolus* Mamiya & Kiyohara, 1972), first recorded and described in Louisiana as *Aphelenchoides xylophilus* [1], is native to North America (USA, Canada and Mexico) [2], [3] and is a serious invasive and destructive species to coniferous forests in countries where it has been introduced. This nematode has been considered the causal agent for pine wilt disease since 1971 [4] being transmitted from tree to tree by wood-inhabiting longhorn beetles that belong mainly to the genus *Monochamus* (Coleoptera: Cerambycidae) [5]. PWN was introduced in Japan at the beginning of the 20^th^ century [6] and later in mainland China [7], Taiwan [8], [9] and Korea [10] which caused massive mortality of native pine trees. PWN was first recorded in Europe (Portugal) in 1999 [11]; later on the Portuguese island of Madeira, 900 km SW from the European continent in 2010 [12]; and more recently in three locations in Spain close to the Portuguese border [13]. The international spread of PWN occurs mainly through the movement of infested logs, untreated wood products and wood-packaging material. To prevent further spread and new introductions, China considers the nematode as a quarantine organism and the European and Mediterranean Plant Protection Organization has placed it on the A2 list (EPPO, http://www.eppo.int). A2 pests are locally present in the EPPO region, and EPPO recommends that its member countries regulate them as quarantine pests. During the 1990 s, a quarantine on green lumber exports to Europe caused an estimated annual loss to the American forest industry of US $100 million [14].

The genus *Bursaphelenchus* currently contains nearly ninety species [15]–[17], which are split into 13 typological groups: namely *abietinus*, *africanus*, *cocophilus*, *eggersi*, *eremus*, *fungivorus*, *hofmanni*, *kevini*, *leoni*, *okinawaensis*, *sexdentati*, *sinensis*, *xylophilus* and one unassigned group [17]. The *xylophilus* group contains *B. baujardi* Walia, Negi, Bajaj & Kalia, 2003; *B. conicaudatus* Kanzaki, Tsuda & Futai, 2000; *B. doui* Braasch, Gu, Burgermeister & Zhang, 2005; *B. firmae* Kanzaki, Maehara, Aikawa & Matsumoto, 2012; *B. fraudulentus* Ruhm, 1956; *B. gillanii* Schönfeld, Braasch, Riedel & Gu, 2013; *B. koreanus* Gu, Wang & Chen, 2013; *B. luxuriosae* Kanzaki & Futai, 2003; *B. masseyi* Tomalak, Worrall & Filipiak, 2013, *B. mucronatus kolymensis* (Korentchenko, 1980) Braasch, Gu, Burgermeister, 2011; *B. mucronatus mucronatus* (Mamiya & Enda, 1979) Braasch, Gu, Burgermeister, 2011; *B. paraluxuriosae* Gu, Wang, Braasch, Burgermeister & Schroder, 2012; *B. populi* Tomalak & Filipiak, 2011; *B. singaporensis* Gu, Zhang, Braasch & Burgermeister, 2005; *B. trypophloei* Tomalak & Filipiak, 2011 and *B. xylophilus* [15]–[20]. Identification of these species using traditional morphology requires a high level of expertise [15]–[17] and can be very time-consuming and inconclusive. However, rapid and accurate identification of PWN is required in order to comply with quarantine regulations and to prevent the movement of PWN between countries. Molecular diagnosis is potentially simple, rapid, sensitive and reliable and could be used to determine with relative certainty the presence of this nematode in wood.

A number of DNA-based tests have been developed to identify PWN with high sensitivity and specificity using a variety of methods including dot-blot analysis [21]; RFLP [22]; PCR-RFLP [23]–[27]; RAPD [28]; PCR by species-specific primers based on internal-transcribed-spacer (ITS) regions [29]–[31]; intergenic spacer [32]; satellite DNA [33]; heat-shock-protein 70 [34]; DNA-topoisomerase-I gene [35]; SCAR [36]; loop-mediated isothermal amplification [37]; and real-time PCR [35], [38]–[44]. Real-time PCR offers an advantage over conventional PCR in that it is generally more sensitive and less time-consuming without post-PCR-agarose-gel electrophoresis. Although these methods are available, none of the tests can be implemented directly in a lab without extensive genomic analysis, lab tests and evaluation on the specificity for a wide range of species.

The Nematode Assay Section of the Agronomic Division of North Carolina Department of Agriculture & Consumer Services (NCDA&CS) is a high-throughput and publicly operated lab. In fiscal year 2013, 3,934 pine-wood samples were analyzed and 233 reports were generated for USDA/APHIS/PPQ in connection with the issuing of phytosanitary certificates for exported pine-wood logs to China. The objective of this study was to characterize the DNA sequences of *Bursaphelenchus* and aphelenchids and develop and validate PWN-specific primers for a reliable, sensitive and rapid real-time PCR assay to support the certification program and diagnostic services. The specificity, sensitivity and application of the assay were demonstrated.

## Materials and Methods

### Nematode Samples

A total of 97 nematode populations with wide-ranging geographical distributions and host species associations were used to evaluate the specificity and sensitivity of this assay (Table 1). They included 45 populations of *B. xylophilus*; 36 populations of 21 other species of *Bursaphelenchus* which belong to the *abietinus*, *cocophilus*, *eggersi*, *fungivorus*, *hofmanni*, *kevini*, *leoni*, *sexdentati*, *xylophilus* groups and one unassigned group from a total of 13 groups in the genus *Bursaphelenchus* [17]; 15 populations of *Aphelenchoides besseyi* Christie, 1942, *A. fragariae* Christie, 1942, *Aphelenchoides* sp. and *Aphelenchus avenae* Bastian, 1865; and one population of mixed nematode species. This mixed population was from a North Carolina centipede lawn and contained *Belonolaimus longicaudatus* Rau, 1958; an unidentified Dorylaimid; *Helicotylenchus* sp.; *Hemicaloosia graminis* Zeng, Ye, Tredway, Martin & Martin, 2012; *Hoplolaimus galeatus* (Cobb, 1913) Thorne, 1935; *Mesocriconema* sp.; an unidentified Rhadtitid; and *Xiphinema americanum* Cobb, 1913. Three species were included from the *xylophilus* group including *Bursaphelenchus xylophilus* from the USA (R-type = no mucro), Canada (R-type, except for 187 as M-type, with mucro), China (M-type) and Japan (R-type); *B. mucronatus kolymensis* from Norway, Finland, Germany and France; *B. mucronatus mucronatus* from Japan; and *B. fraudulentus* from Hungary.

**Table 1:** Nematode species, group in *Bursaphelenchus* and populations, gene sequenced, GenBank accession number and real-time-PCR result.

Species (group)	Sample No.	Locality	Host		GenBank Accession Number			Threshold Cycle (C_t_)by PWN*-*SpecificPrimer/Probe	C_t_ by Nematode-Universal Primer/Probe
				SSU	LSU	mtCOI	ITS		
*Bursaphelenchus abietinus* (*abietinus* group)	137	Austria	*Abies alba*	AY508011	AY508074	AY508037		0	23.00
*B. abruptus* (unassigned group)	136	MD, USA	*Anthophora abrupta*	AY508010	AY508073	AY508036		0	31.22, 29.18^a^
*B*. *anatolius* (*kevini* group)	170	Turkey	*Halictus* sp.	AY508025	AY508093	AY508056		0	24.89
*B. borealis* (*leoni* group)	138	Germany	*Picea abies*	AY508012	AY508075	AY508038		0	22.41
*B. cocophilus* (*cocophilus* group)	140	Costa Rica	*Elaeis guineensis*		AY508076			0	27.07
	144	Honduras	*Cocos nucifera*	AY509153	AY508077	AY508039		0	26.31
*B. eggersi* (*eggersi* group)	146	Germany	*Pinus sylvestris*	AY508013	AY508078	AY508040		0	12.48
*B. fraudulentus* (*xylophilus* group)	148	Hungary	*Quercus* sp.		AY508079	AY508042		0	17.98
	150	Russia	*Larix* sp.	AY508014	AY508080	AY508043		0	22.28
	151	Germany	*Picea/Pinus*	AY508015	AY508081	AY508044		0	27.26
*B. fungivorus* (*fungivorus* group)	153	Germany	Greenhouse soil	AY508016	AY508082	AY508045		0	27.07
*B*. *gerberae* (*hofmanni* group)	169	Trinidad	*Cocos nucifera*	AY508024	AY508092	AY508055	KF025320	0	27.96
*B. hellenicus* (*abietinus* group)	154	Greece	*Pinus brutia*	AY508017	AY508083	AY508046		0	31.9429.97
*B. hofmanni* (*hofmanni* group)	155	Germany	*Picea abies*	AY508018	AY508084	AY508047		0	23.00
*B. hylobianum* (*abietinus* group)	160	Russia	*Larix sibirica*	AY508019	AY508085	AY508048		0	24.38
*B. kevini* (*kevini* group)	355	Santa Cruz Island, CA, USA	*Halictus farinosus*		AY753532	AY753533		0	24.44
	356	Santa Cruz Island, CA, USA	*Halictus farinosus*			EU325687		0	22.47
*B. mucronatus kolymensis* (*xylophilus* group)	164	Norway	*Pinus sylvestris*		AY508087	AY508050		0	21.70
	165	Finland	*Pinus sylvestris*	AY508021	AY508088	AY508051	KF025329	0	12.66
	166	Germany	*Picea abies*		AY508089	AY508052		0	13.95
	167	Germany	*Pinus sylvestris*	AY508022	AY508090	AY508053		0	29.08, 29.97, 32.71
	168	Germany	*Picea abies*	AY508023	AY508091	AY508054	KF025318	0	24.72, 32.54, 23.04
	N7	France	Wood packaging material					0	20.81, 22.90
*B. mucronatus mucronatus* (*xylophilus* group)	163	Japan	Pine tree	AY508020	AY508086	AY508049		0	30.50
*B. paracorneolus* (*hofmanni* group)	172	Germany	*Picea abies*	AY508027	AY508095	AY508058		0	29.19, 32.13
*B*. *platzeri* (*cocophilus* group)	171	CA, USA	*Carpophilus humeralis*	AY508026	AY508094	AY508057		0	28.16
*B. poligraphi* (*sexdentati* group)	173	Germany	*Picea abies*	AY508028	AY508096	AY508059		0	30.30, 27.19
*B. rufipennis* (*hofmanni* group)	727	WI, USA	Spruce bark beetle (*Dendroctonus rufipennis*) from *Picea* sp.					0	22.17
*B. seani* (*fungivorus* group)	174	CA, USA	*Anthophora bomboides*		AY508097	AY508060		0	25.25
	175	CA, USA	*Anthophora bomboides*	AY508029	AY508098	AY508061		0	31.47
	176	CA, USA	*Anthophora bomboides*	AY508030	AY508099	AY508062		0	24.7
*B. sexdentati* (*sexdentati* group)	177	Greece	*Pinus nigra*		AY508100	AY508063		0	25.35 31.46
	178	Greece	*Pinus nigra*		AY508101	AY508064		0	24.87
	179	Greece	*Pinus radiata*	AY508031	AY508102	AY508065		0	23.93
	180	Italy	*Pinus pinaster*	AY508032	AY508103	AY508066		0	29.08
*B. tusciae* (*eggersi* group)	183	Italy	*Pinus pinea*	AY508033	AY508104	AY508067		0	27.68
*B. xylophilus* (*xylophilus* group)	185	Canada	*Pinus banksiana*		AY508105	AY508068	KF025325	15.98	16.36
	186	Japan	*Pinus densiflora*	AY508034	AY508106	AY508069	KF025326	10.14, 13.37	15.18
	187	NB, Canada	Pine tree		AY508107	AY508070	KF025327	10.53, 8.69, 12.02	12.80
	188	QC, Canada	*Pinus/Picea*		AY508108	AY508071	KF025328	10.27	14.97
	345	MO, USA	*Pinus sylvestris*				KF025324	24.62	29.18
	N18	China	*Pinus kesiya*					27.86, 27.93	28.73, 25.16
	2008-00610	USA	Pine tree					30.94	32.94
	2008-01182	Swansboro, NC, USA	Pine tree				KF025321	24.34	32.94, 24.76
	2008-12108	USA	Wood chips				KF025323	28.61, 28.30	27.16, 27.51, 26.95
	2008-12140	USA	Wood chips				KF025321	24.86, 26.81	26.19
	2008-26471	USA	Wood chips				KF025323	24.91, 30.85	28.95
	2008-26479	USA	Wood chips				KF025321	24.95	28.3
	2008-26522	USA	Wood chips				KF025321	25.56, 26.62, 23.06	26.61, 24.59
	2009-00185	USA	Wood chips				KF025321	22.57	23.27
	2009-00211	USA	Wood chips				KF025321	30.21	27.99
	2009-00251	USA	Wood chips				KF025321	20.40, 20.96	21.00
	2009-00740	USA	Pine tree				KF025321	21.91, 22.54, 23.55	22.28
	2009-01010	USA	Wood chips				KF025321	28.81	23.58
	2009-01042	Beaufort, NC, USA	Pine tree				KF025321	21.24, 28.56	22.77
	2009-12070	USA	Wood chips				KF025321	24.56	27.43
	2009-23917	USA	Pine tree				KF025321	22.33	25.05
	2010-00489	Beaufort, NC, USA	Japanese black pine (*Pinus thunbergii*)				KF025321	24.83, 25.61	31.98
	2010-00695	Wilmington, NC, USA	Japanese black pine (*Pinus thunbergii*)	KF025317			KF025321	24.14	24.56
	2012-05740	Emerald Isle, NC, USA	Japanese black pine (*Pinus thunbergii*)				KF025321	20.86, 22.88	22.40
	2012-08812	Seven Spring, NC, USA	Pine-wood log				KF025321	28.81	22.87
	2012-19124	Atlantic Beach, NC, USA	Japanese black pine (*Pinus thunbergii*)			KF025330	KF025322	20.86, 21.91, 22.04, 23.58,23.95, 25.97	23.26, 23.96, 24.95, 25.80
	2012-33423	USA	Pine-wood log				KF025321	24.22, 26.61	26.63, 31.32
	2012-33666	USA	Pine-wood log				KF025323	24.21	24.58
	2012-34017	USA	Pine-wood log				KF025323	28.00	22.67
	2013-00850	USA	Pine-wood log				KF025323	24.49	22.82
	2013-00930	USA	Pine-wood log	KF025319			KF025321	21.96	21.49
	2013-02091	USA	Pine-wood log				KF025321	22.84	22.58
	2013-02478	USA	Pine-wood log				KF025321	29.96	26.18
	2013-03266	USA	Pine-wood log				KF025321	30.40	30.19
	2013-04587	USA	Pine-wood log				KF025323	28.53	30.71
	2013-09254	Onslow County, NC, USA	Japanese black pine (*Pinus thunbergii*)				KF025323	22.60, 23.86, 23.98	25.96, 26.37, 30.91
	2013-33795	USA	Pine-wood log (*Pinus strobes*)					28.74, 28.93, 29.89	29.81
	2013-34160	USA	Pine-wood log					23.91, 25.04, 28.94	23.34
	2013-34252	USA	Pine-wood log					27.77	28.64
	2013-34362	USA	Pine-wood log					27.39	27.86
	2013-34252	USA	Pine-wood log					^b^	^b^
	2013-34362	USA	Pine-wood log					^b^	^b^
	2013-34693	USA	Pine-wood log					^b^	^b^
	2013-34814	USA	Pine-wood log					^b^	^b^
	2013-34973	USA	Pine-wood log (*Pinus strobes*)					^b^	^b^
*Aphelenchoides besseyi*	98	FL, USA	Strawberry (*Fragaria ananassa*)	AY508035	AY508109	AY508072		0	23.77, 30.44
*Aphelenchoides fragariae*	2010-02011	Raleigh, NC, USA	Ornamental plant					0	22.39
	2010-02016	Raleigh, NC, USA	Ornamental plant					0	21.65
	2012-08056	Raleigh, NC, USA	Fern (*Woodsia obtusa*)					0	27.91, 29.14
	M112	Raleigh, NC, USA	Lantana					0	31.67
*Aphelenchoides* sp.	757	NC, USA	Soil around pine tree					0	22.04, 30.18
	2008-01707	USA	Pine tree					0	27.83
	2010-00129	Crane, IN, USA	Pine-wood-packaging material					0	22.37
	12-6370	Crane, IN, USA	Pine-wood-packaging material	KF032031	KF032032		KF032031	0	29.19
	12-23697	USA	Pine-wood log					0	29.67, 31.05
	12-32618	USA	Pine-wood log					0	30.18
	2012-33345	McAlester, OK, USA	Pine-wood-packaging material	KF032030			KF032030	0	22.18, 24.52, 24.96
	2013-34187	USA	Pine-wood log					^b^	^b^
	2013-34814	USA	Pine-wood log					^b^	^b^
*Aphelenchus avenae*	103	FL, USA	Soil from roots of *Ilex vomatoria*					0	21.97
Mixed soil nematode species	2013-33843	New Bern, NC, USA	Centipede grass					0	32.99

a : Multiple C_t_ values were from various replicates.^ b^: Duplex real-time PCR only, see results in table 4.

The PWN samples used were extracted from exported pine-wood logs, wood chips, wood-packaging materials and declining pine trees, but other species were from various sources as indicated in Table 1. USDA/APHIS/PPQ officers followed the protocol provided in their Export Program Manual (http://www.aphis.usda.gov/import_export/plants/manuals/domestic/downloads/xpm.pdf) when inspecting and sampling PWN in pine-wood logs slated for export. The number of units to inspect was based on one of two hypergeometric tables at 95% confidence of detecting a 10% or 5% infestation with 100% efficacy, depending on the state. Two holes, up to six inches (15 cm) deep, were drilled per log at six inches (15 cm) from both ends using a 2.125-inch (5.4-cm), self-feeding-wood bit. The wood shavings from two logs were mixed together, and a minimum 200 g of wood shavings were collected as one lab sample. Samples were shipped overnight to NCDA&CS for nematode analysis. Some nematode samples were collected from Europe, North America, Central America and Asia. They were reared on cultures of the fungus *Monilinia fructicola* on potato dextrose agar plates, except for *Bursaphelenchus cocophilus* (Cobb, 1919) Baujard, 1989, which was extracted from infested hosts and killed and shipped in 95% ethanol before subsequent DNA extraction.

### Extraction of Nematodes

Each wood sample was weighed and assigned a unique lab ID number. The wood shavings for each sample were placed in a single layer inside a wire basket lined with a large, single-folded Kimwipe (37 cm×42 cm, Kimberly-Clark Professional, Neenah, WI, USA) and completely wrapped. The baskets were then placed into plastic containers (36 cm L×24 cm W×14 cm H). Tap water was added until the wood shavings were completely submerged. After incubation for 24 hours at room temperature to allow nematodes to move out [45], the wood-containing baskets were removed gently and the supernatant water was vacuumed out slowly using a H_2_O Pro electrical pump (#50AC110B, FM Industries, Milwaukee, WI) [45]. Then the remaining nematode suspension was left to settle for 30 minutes at a slant, approximately 45 degrees, after which additional supernatant water was vacuumed. Approximately 100 ml of the remaining nematode solution was decanted into beakers and allowed to settle for 30 minutes. The supernatant water was then vacuumed with a water-faucet-vacuum-aspirator apparatus to approximately 20 ml. No sieve was used in the nematode extraction to avoid cross contamination between samples. The sample was poured into a counting dish (7.5 cm L×3 cm W×1.5 cm H), and the nematodes present were identified and counted under a Nikon Diaphot 200 inverted microscope (Tokyo, Japan). Further species confirmation was performed with a Leica DM2500 compound microscope (Leica Microsystems Inc., Buffalo Grove, IL) with interference contrast up to 1,000× magnification.

### DNA Preparation

One to ten nematodes were transferred to a glass microscope slide (7.5 cm x 2.5 cm), squashed using a pipette tip in about 5 µl of AE buffer (10 mM Tris-Cl, 0.5 mM EDTA, pH 9.0), and then placed in a 1.5-ml microtube. AE buffer was added up to 50 µl. DNA extracts were stored at -20°C until used as PCR template.

### Polymerase Chain Reaction (PCR) and DNA Sequencing

**Figure 1:**
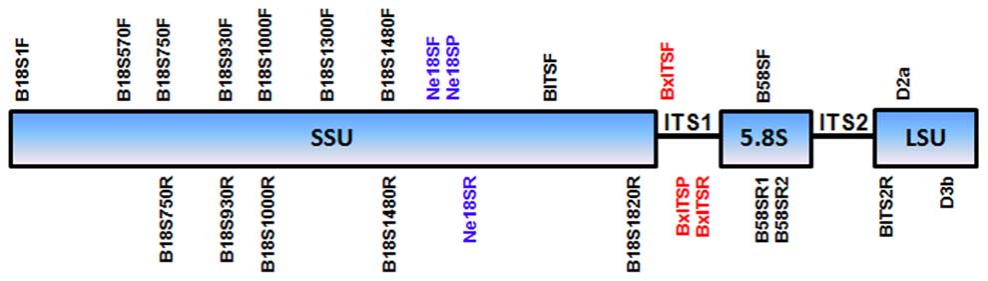
Primer and probe locations for PCR amplification, sequencing and real-time PCR of ribosomal DNA.

PCR for ribosomal DNA near-full-length-small subunit (SSU), ITS and cytochrome-oxidase-gene subunit I (mtCOI) amplification was conducted using various combinations of universal forward and reverse primers (Table 2). These primers were based on the conserved sites from a multiple alignment of many *Bursaphelenchus* species and some aphelenchids from GenBank and their approximate positions were shown in Fig. 1. The primer selection criteria were as follows: Tm (melting temperature) 55 to 60°C, primer length 18 to 22 bp, and absence of secondary structure when possible. Primers for partial ribosomal-DNA-large-subunit D2/D3 (LSU D2/D3) were forward-primer D2a (5′ ACAAGTACCGTGAGGGAAAGT 3′) and reverse-primer D3b (5′ TGCGAAGGAACCAGCTACTA 3′) [46]. The 25-µl PCR was performed using Apex-Taq-red-master-mix DNA polymerase (Genesee Scientific Corporation, San Diego, CA, USA) according to the manufacturer’s protocol in a Veriti® thermocycler (Life Technologies, Carlsbad, CA). The thermal cycler program for PCR was as follows: denaturation at 95°C for 5 min, followed by 40 cycles of denaturation at 94°C for 30 s, annealing at 55°C for 45 s, and extension at 72°C for 1 min. A final extension was performed at 72°C for 10 min. PCR products were cleaned using ExoSap-IT (Affymetrix, Inc., Santa Clara, CA, USA) according to the manufacturer’s protocol and were sequenced by Genomic Sciences Laboratory in North Carolina State University using a 3730 XL DNA Analyzer (Life Technologies, Carlsbad, CA). The molecular sequences were compared with other nematode species available at the GenBank sequence database using the BLASTn homology search program. The sequences were deposited into GenBank database.

**Table 2:** PCR Primers and Real-Time PCR Primers and Probes.

No.	Primer	Gene	Direction^a^	Sequence
1	B18S1F^b^	SSU	F	ATACGCATGTCTAAGTGGAG
2	B18S570F	SSU	F	AAGTCTGGTGCCAGCAGCC
3	B18S750F	SSU	F	GCAGGATTACTTTGAACGGCTC
4	B18S750R	SSU	R	GAGCCGTTCAAAGTAATCCTG
5	B18S930F	SSU	F	AATTCGTGGACCGTAGCGAG
6	B18S930R	SSU	R	CTCGCTACGGTCCACGAATT
7	B18S1000F	SSU	F	GTCAGAGGTTCGAAGGCG
8	B18S1000R	SSU	R	CGCCTTCGAACCTCTGAC
9	B18S1300F	SSU	F	GCATGGCCGTTCTTAGTTCGT
10	B18S1480F	SSU	F	GGCCGCACGCGTGCTACAAT
11	B18S1480R	SSU	R	ATTGTAGCACGCGTGCGGCC
12	B18S1820R	SSU	R	CTACGGCTACCTTGTTACGAC
13	BITSF	ITS	F	ATCGCAGTGGCTTGAACCGG
14	B58SF	ITS	F	AATCGCAGTGAATTGCGATA
15	B58SR1	ITS	R	CTCATAATATCTGTAATTCGTAC
16	B58SR2	ITS	R	AACACACCCTGAATC
17	BITS2R	ITS	R	TCCTCTGCTTACTGATATGC
18	BCOIF	mtCOI	F	GGTGGTTTTGGTAATTG
19	BCOIR	mtCOI	R	ACAACCAATTAAACCAAT
20	Ne18SF	SSU	F	ATTGACGGAAGGGCACCAC
21	Ne18SP	SSU	F	5′−/5HEX/TGCGGCTTA/ZEN/ATTTGACTCAACACGGG/3IABkFQ/−3′
22	Ne18SR	SSU	R	GAACGGCCATGCACCAC
23	BxITSF	ITS1	F	GATGGCGGTTCGATTCGCG
24	BxITSP	ITS1	R	FAM AACTCAACAACAGCACGTAGA MGBNFQ
25	BxITSR	ITS1	R	TGGCTGGTCTCATCTGTCGG

a : F: forward, R: reverse. ^b^: number after 18S represents the relative primer position in rDNA SSU gene.

### Real-time PCR

**Figure 2:**
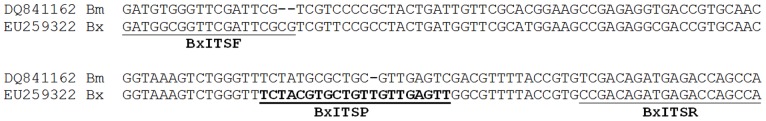
Primer (BxITSF, BxITSR) and probe (BxITSP) design based on the multiple alignment of ITS1 DNA sequences of *Bursaphelenchus* species. Data only show two representative species, *B. xylophilus* (EU259322) and *B. mucronatus* (DQ841162).

A florescent probe (BxITSP) specific for PWN targeting ITS1 was labeled with reporter dye 6-carboxy-fluorescein (FAM) (518 nm maximum emission) at the 5′ end, and the 3′ end was modified with nonfluorescent quencher (NFQ) (Table 2). The forward and reverse primer sequences (BxITSF, BxITSR) yield an amplicon of 140 bp. The design of this probe and these primers (BxITSF, BxITSP, BxITSR) is based on a multiple alignment of ITS1 sequences of some representative species of *Bursaphelenchus* species from GenBank and our sequences. These sequences included all 13 species in the *xylophilus* group, except *B. baujardi.* The 21-bp probe sequence is PWN-specific, providing 100% identity with 100% coverage for 52 PWN sequences from GenBank using BLASTn search but low matches for all other nematode species included all available species in the *xylophilus* group. Fig. 2 shows the alignment and priming sites of two representative sequences of PWN (EU259322) and the sister species of *B. mucronatus* (DQ841162). Primer Express 3.0 from Life Technologies was used for primer and probe design. These primers and the probe were synthesized by Life Technologies and prepared in 10× working solution by mixing 18 µl of 100-µM forward primer, 18 µl of 100-µM reverse primer, 5 µl of 100-µM probe and 159 µl of 1× TE buffer.

The second nematode-universal primer/probe set (Ne18SF, Ne18SP and Ne18SR) was designed based on the conserved sites of SSU from a multiple alignment of 54 species of nematodes in the genera *Anguina*, *Aphelenchoides*, *Aphelenchus*, *Ascaris, Bursaphelenchus*, *Caenorhabditis, Cephalobus, Cryptaphelenchus*, *Ditylenchus*, *Ektaphelenchus*, *Globodera*, *Howardula*, *Laimaphelenchus*, *Longidorus*, *Meloidogyne*, *Myolaimus, Pristionchus, Ruehmaphelenchus*, *Schistonchus* and *Seinura* as an internal positive control. The double-quenched probe Ne18SP is labeled with a different dye HEX (536-nm maximum emission) to allow for duplex-real-time PCR. Real-time PCR SciTool (PrimeQuest and OligoAnalyzer) by Integrated DNA Technologies, Inc. (Coralville, IA, USA) (http://www.idtdna.com/Scitools/Applications/RealTimePCR/) was used for primer and probe design. The primers amplify a 142-bp region of ribosomal DNA SSU for a variety of nematode species. These primers and probe were synthesized by Integrated DNA Technologies, Inc. and prepared in 10× working solution.

The 10-µl real-time PCR contained 5 µl of 2× TaqMan® real-time PCR master mixes (Life Technologies), 1 µl of 10× primer and probe mix, 3-µl water and 1-µl-DNA template. This provides for 900 nM of each primer and 250 nM of the probe at the final 1× concentration. A two-step, thermal-cycling program was used: denaturation at 95°C for 10 min, followed by 40 cycles of denaturation at 95°C for 15 s, annealing and extension at 60°C for 1 min in Applied Biosystems® 7500 Real-Time PCR Systems (Life Technologies).

One sample (2013–33795) with three life stages (single female, single male and single juvenile) in separate tubes was prepared in 50-µl AE buffer, and a 5-, 10- and 20-fold dilution was prepared and tested for the sensitivity of the real-time PCR assay by PWN-specific primer and probe mix.

### Duplex-Real-time PCR

The real-time PCR contained 5 µl of 2× TaqMan® real-time PCR master mixes, 1 µl of 10×-PWN-specific primer and probe mix, 1 µl of 10× nematode-universal primer and probe mix, 2-µl water and 1-µl-DNA template. The same two-step, thermal-cycling program was used.

## Results and Discussion

### Morphological Identification of PWN

**Figure 3:**
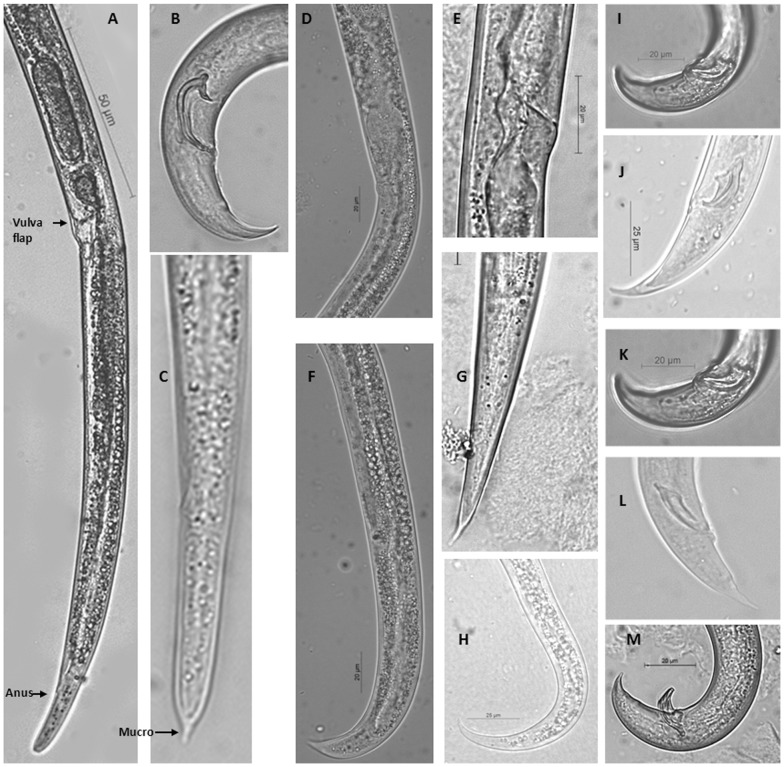
Morphological comparisons between *Bursaphelenchus xylophilus* (A-C) and other wood-inhabiting aphelenchids (D-M). A. Posterior female end showing vulva flap, anus and blunt tail. B. Male spicule and tail end. C. Female tail showing mucro. D-E. Vulva without flap. F-H. Female pointed tail. I-M. Spicule and tail end of male.

PWN is a gonochoristic species that can be typologically characterized by the presence of a vulva flap and broad tail with rounded tip in the female, and large, arcuate spicules in the male that are trapezoidal in lateral view, with a sharply pointed prominent rostrum and cucullus (disc-like projection) at the distal ends. Other characters and measurements are available from the original description by Mamiya & Kiyohara [5] and EPPO diagnostic standard [45]. A quick guide for distinguishing PWN from other wood-inhabiting aphelenchids is presented in Fig. 3. Typological species identification is not so challenging for wood logs and products for export from the USA because none of the other closely related species in the *xylophilus* group are known to be present in the USA.

### DNA Sequencing

The ribosomal DNA SSU, LSU D2/D3, ITS and mitochondrial-DNA COI were sequenced, and their accession numbers from GenBank are presented in Table 1. Some of data were collected from our previous study [47]. Sequencing analysis revealed PWN has unique sequences in all these markers and is closest to its sister species *B. mucronatus*. Molecular phylogenetic relationships of *Bursaphelenchus* species are available in Ye et al. [47] and Kanzaki et al. [20]. The multiple sequence alignment revealed the protein-coding-gene mtCOI has no insertion/deletions, but only site variations, except for *B. cocophilus* with a 3-bp deletion. SSU and LSU D2/D3 have few insertions/deletions and some site variations, and ITS is the most variable with considerable insertions/deletions and site variations. Therefore, ITS1 was chosen as the real-time-PCR marker to ensure the specificity. The primer/probe design based on the variable sites is shown in Fig. 2.

### Real-time PCR

**Figure 4:**
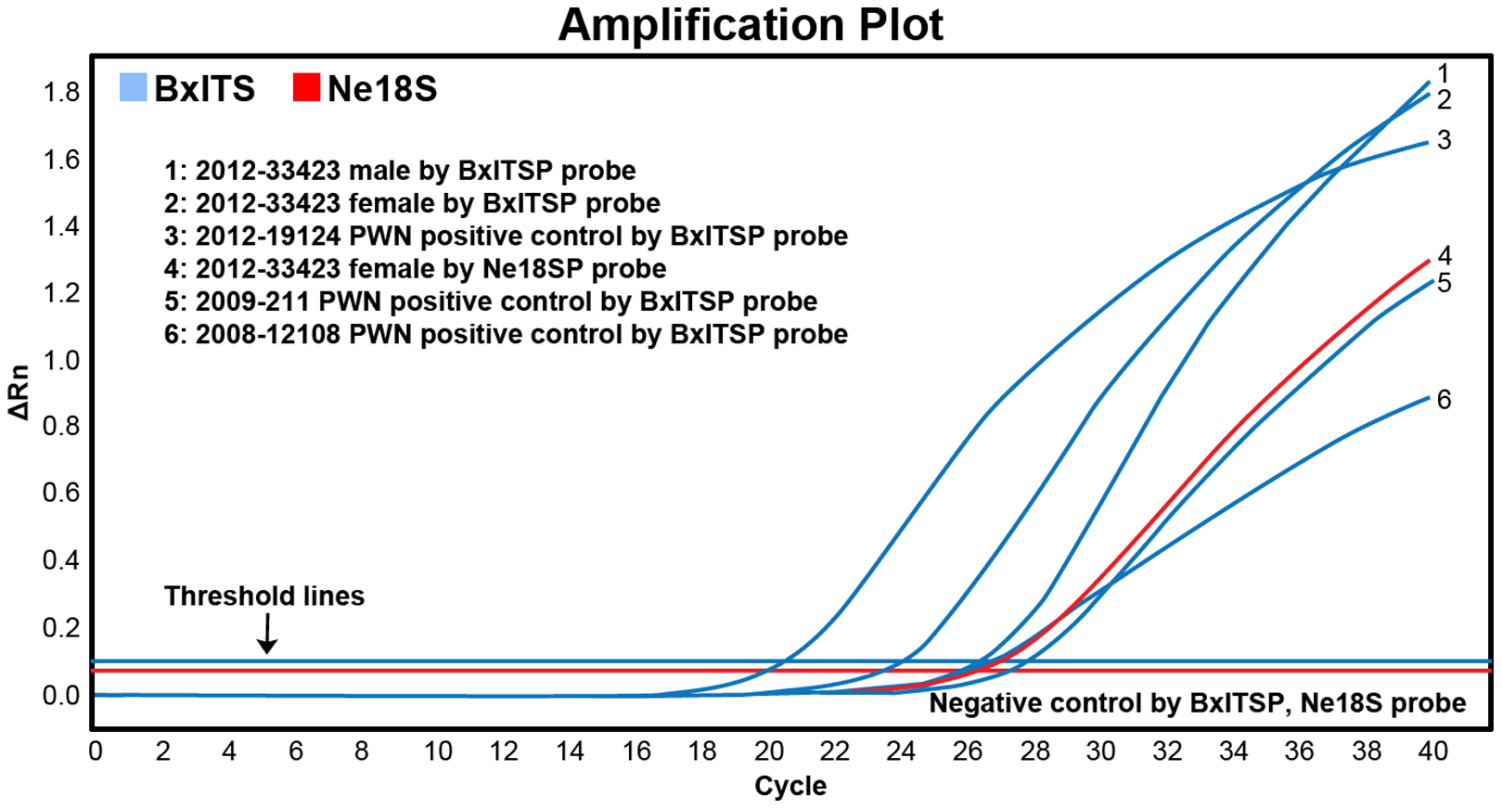
Example of a real-time-PCR result for testing sample 2013–33423 by PWN-specific- and nematode-universal- primer/probes.

Using the PWN-specific-primer/probe set, all assays were 100% specific and accurate for the detection of PWN with a FAM threshold cycle (C_t_ value) from 8.69–30.94. No fluorescent signals were obtained for samples other than PWN. The nematode-universal primer/probe set showed all PWN samples and all other non-PWN samples to be positive for the presence of nematode SSU with HEX C_t_ from 12.48–32.99 (Table 1). This nematode-universal marker also works for a number of mixed species from the soil nematode sample (2013–33843) from a centipede lawn. Fig. 4 is the amplification plot of an example assay to test sample 2012–33423. In this assay, samples 2008–12108, 2009–00211 and 2012–19124, which were previously identified as PWN, were used as positive controls. Water was used as the negative control. This assay revealed the single female and single male of 2012–33423 were positive using PWN-specific primer/probe, and the single female was positive using the nematode-universal primer/probe. The fact that all three positive controls were positive and the negative control was negative demonstrates that the reactions were successful and valid. If a sample tested PWN-negative, it then should be nematode-positive to be considered a valid assay. If it tested nematode-negative, the nematode DNA preparation and/or real-time PCR should be repeated. In any assay, the multi-component plot was examined to ensure the reaction was set up correctly and the reaction mix had not evaporated during the approximately 90-minute-long-PCR amplification.

**Figure 5:**
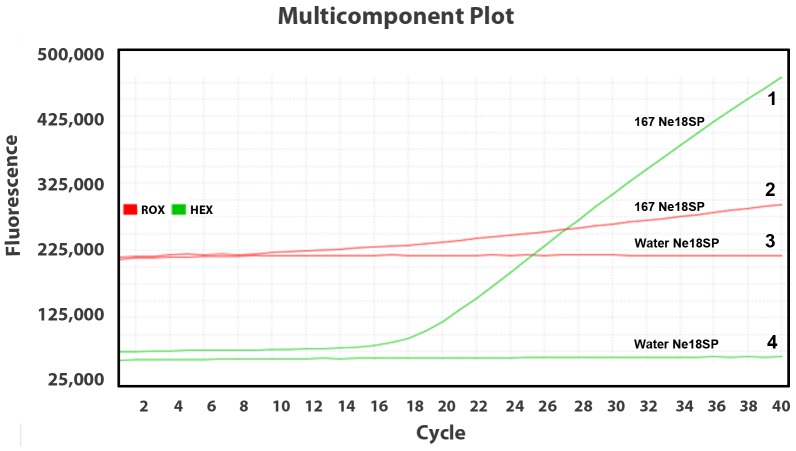
Multi-component plot of a real-time-PCR result. This plot shows increased reference dye ROX (Curve 2) and non-increased ROX (Curve 3), increased reporting dye HEX (Curve 1) and non-increased HEX (Curve 4) for *Bursaphelenchus mucronatus* (sample 167, Curves 1 and 2) and negative control (Curves 3 and 4).

Fig. 5 is an example of a test showing one sample 167 (curves 1 and 2) and negative control (curves 3 and 4) using the nematode-universal primer/probe. The starting fluorescence of ROX in red (curves 2 and 3) were close to each other at *ca* 220,000, and the starting fluorescence of HEX in green (curves 1 and 4) was *ca* 74,000. This revealed the pipetting for adding 2× TaqMan® real-time PCR master mixes containing background-dye ROX and adding primer/probe containing reporting-dye HEX was approximately even. At the end of the amplification at cycle 40, the ROX (curve 2) on sample 167 had increased to 250,000, indicating evaporation in the reaction tube due to problems with the cap seal. The ROX (curve 3) on the negative control remained the same, demonstrating that no evaporation occurred. The reporting-dye HEX (curve 1) on sample 167 increased considerably to 475,000, indicating a positive result. Lack of amplification in the negative control as indicated by the HEX (curve 4) corroborates the validity of the test. In this assay, although the reaction tube of sample 167 was not completely enclosed, the amplification of a positive result was still considered successful and valid, and the fluorescent signal of HEX could be normalized automatically by the reference to the ROX dye in the amplification plot. In any test, the multi-component plot should be reviewed and all ROX curves in a test should be without an increase ideally, and any test with odd results should be repeated.

**Figure 6:**
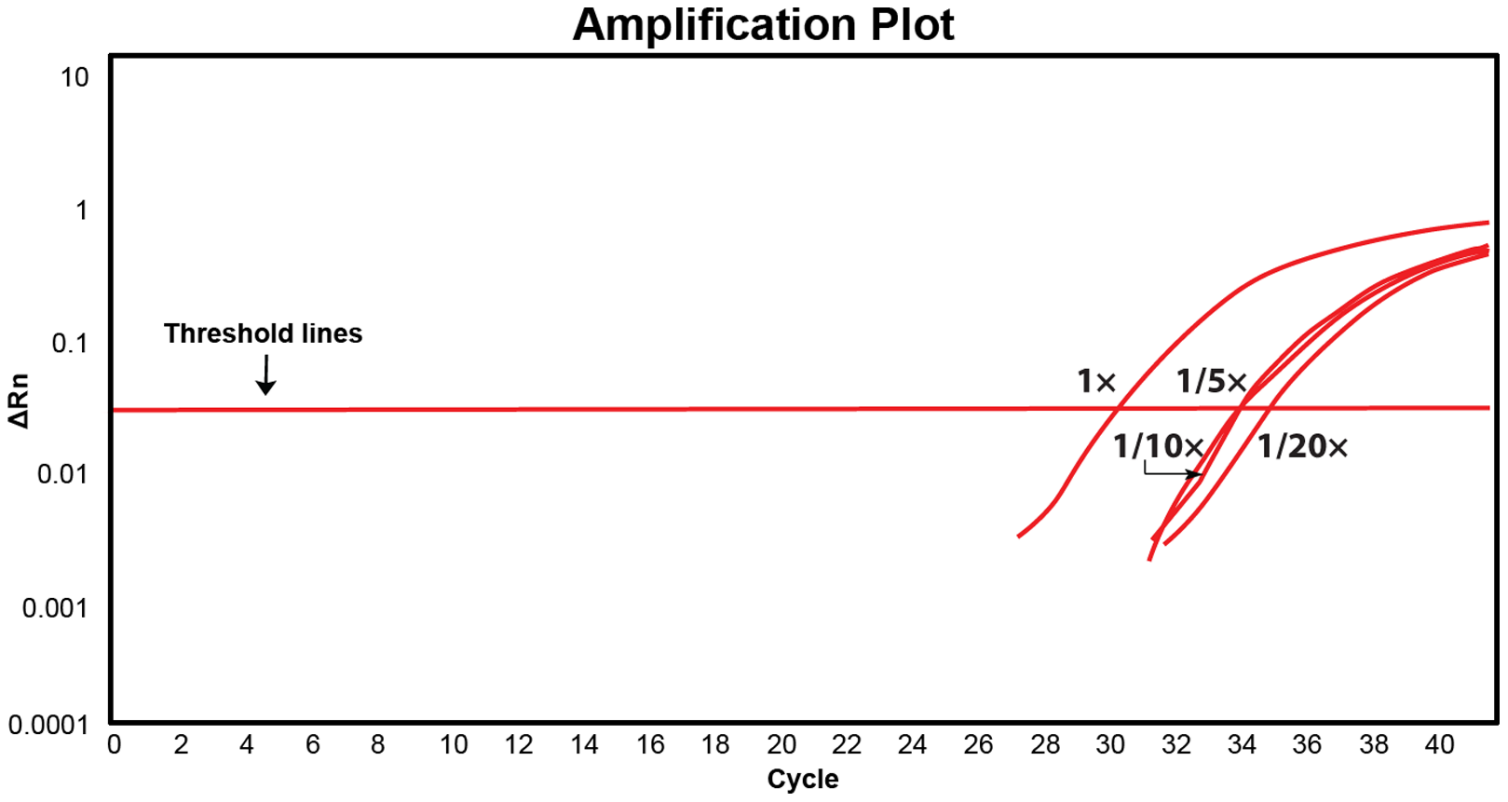
Amplification plot of a real-time-PCR result with different dilutions of a male of *Bursaphelenchus xylophilus* (2013–33795).

The use of the PWN-specific primer/probe with real-time PCR yielded similar results regardless of the life stages used (Table 3). The test could detect a single nematode with a tiny amount of nematode DNA, demonstrated by testing 1/5, 1/10 and 1/20× dilutions, but the dilution extended the C_t_ value up to three cycles when diluted to 1/20× (Fig. 6, Table 3). This result revealed the real-time PCR is highly sensitive, i.e., it can detect a 1-µl nematode template even if a single nematode was squashed and dissolved in 1,000 µl of buffer. This highly diluted DNA template is sufficient to run many molecular tests and replicates. In a real-world application, a single nematode would always be available for preparation in 50 µl buffer or even up to 1,000 µl buffer for real-time-PCR assay.

**Table 3:** Real-time PCR results for nematode sample no. 2013–33795 with different life stages and dilutions.

Life stage		Dilution		
	**1×**	**1/5×**	**1/10×**	**1/20×**
1 female	28.93	29.34	32.83	32.40
1 male	29.89	33.28	33.24	33.99
1 juvenile	28.74	30.40	29.63	31.06

### Duplex-Real-time PCR

**Figure 7:**
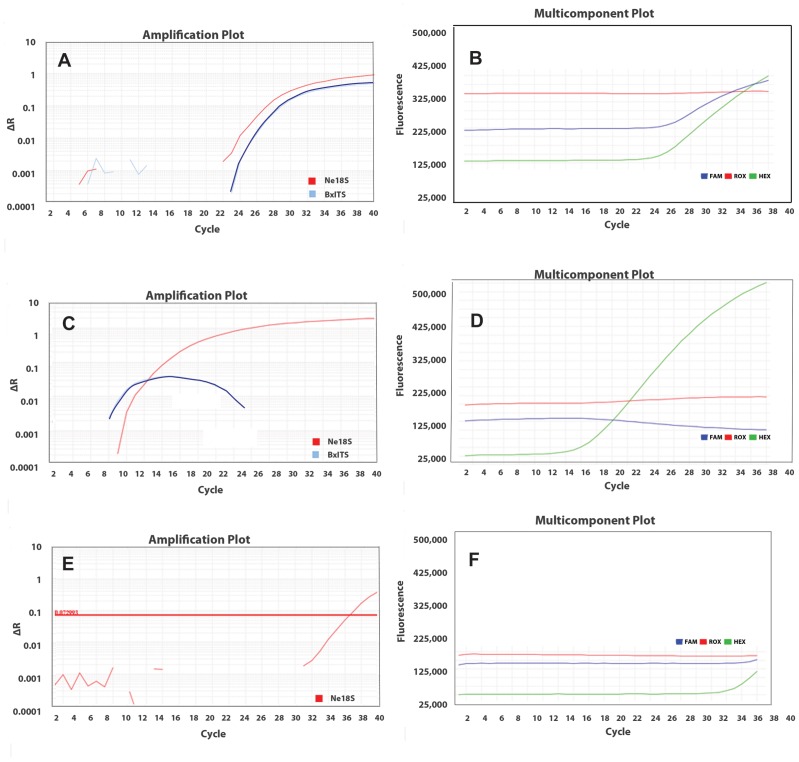
Duplex real-time-PCR result. A. A positive result with two sigmoid FAM and HEX curves in amplification plot. B. A positive result with two sigmoid FAM and HEX curves and non-increased ROX in multi-component plot. C. A negative result with one sigmoid HEX curve, and non-increased FAM curve and non-increased ROX in amplification plot. D. A negative result with one sigmoid HEX curve, and non-increased FAM curve and non-increased ROX in multi-component plot. E. A false-positive result with a slightly later-increased HEX curve (C_t_>35) in amplification plot. F. A false-positive result with a slightly later-increased HEX curve (C_t_>35) and non-increased FAM and ROX curves in multi-component plot.

Duplex-real-time PCR was performed on a subset of samples including 12 nematode species and 15 populations of PWN from a single nematode (female, male or juvenile) up to 20 nematodes (Table 4). All samples were positive regardless of the nematode species with the internal-positive-control marker using the nematode-universal primer/probe, but only positive for PWN samples using the PWN-specific primer/probe (Table 4). A positive amplification in duplex-real-time PCR is represented by two sigmoid curves (PWN dye and nematode dye) in the amplification plot (Fig. 7A) and two sigmoid curves (PWN dye and nematode dye) and no increase in the ROX curve (Fig. 7B) in a multi-component plot. A negative amplification in duplex-real-time PCR is represented by one sigmoid curve (nematode dye) as an internal-positive control, but no amplification in PWN dye in the amplification plot (Fig. 7C) and one sigmoid curve (nematode dye), no increase in the ROX curve and no increase in the PWN curve (Fig. 7D) in the multi-component plot. This duplex PCR further confirmed that the assay is sensitive to any stage of nematode and is sensitive to a single nematode. In a few cases, even in a negative control, a later amplification was observed with C_t_ greater than 35 (Fig. 7E), but the amplification of the reporting dye was not strong (Fig. 7F). This false-positive result is probably due to primer-dimmer formation and/or degradations of the probe.

**Table 4:** Nematode duplex real-time PCR results.

Species	Sample No.	Number of Nematode and Life Stage	Threshold Cycle (C_t_) by PWN*-*Specific Primer/Probe	C_t_ by Nematode-Universal Primer/Probe
*Bursaphelenchus abruptus*	136	10 nematodes	0	31.23
*B*. *anatolius*	170	10 nematodes	0	27.83
*B. fraudulentus*	150	10 nematodes	0	26.47
*B*. *gerberae*	169	10 nematodes	0	30.44
*B. hylobianum*	160	10 nematodes	0	27.33
*B. mucronatus*	163	1 female	0	31.60
	167	1 female	0	31.64
	168	1 female	0	27.16
*B. paracorneolus*	172	10 nematodes	0	30.12
*B. tusciae*	183	10 nematodes	0	30.00
*B. xylophilus*	345	1 male	31.99	29.44
	2009-00251	10 nematodes	23.90	23.09
	2009-00740	10 nematodes	23.06	22.98
	2010-00489	5 nematodes	26.06	26.23
	2009-01010	6 nematodes	32.33	32.16
	2012–19124	1 juvenile	26.25	26.35
		10 nematodes	24.28	24.96
	2012–33423	1 female	29.95	26.79
	2013–09254	20 nematodes	27.64	26.66
	2013–33795	1 male	30.45	31.04
	2013–33795	7 nematodes	25.02	25.55
	2013–34160	3 nematodes	23.91	23.34
	2013–34252	2 nematodes	27.84	25.57
	2013–34362	7 nematodes	26.92	24.95
	2013–34693	10 nematodes	25.94	25.23
			22.99	22.18
	2013–34814	1 female	30.74	27.91
	2013–34973	6 nematodes	26.43	25.08
*Aphelenchoides besseyi*	98	10 nematodes	0	30.37
*Aphelenchoides* sp.	2013–34187	1 female	0	28.96
	2013–34814	1 female	0	28.68
Mixed species	2014–33843	Many nematodes	0	26.13

In conclusion, this study characterized DNA sequences on ribosomal DNA LSU, SSU D2/D3, ITS and mtCOI on a wide range of species in *Bursaphelenchus* and other aphelenchids. Universal primers were developed to perform DNA sequencing on this group of nematodes. Through extensive DNA analysis of these genes, ITS1 was chosen as the marker to develop PWN-real-time PCR. All assays were highly robust and specific for detection of PWN and sensitive for a single nematode regardless of the life stage. This real-time-PCR assay has been successfully applied in export and diagnostic assay services in a high-throughput-nematode-assay lab. One nematode prepared in 50 µl of DNA template provided sufficient material for molecular diagnosis. It is extremely sensitive even when a single nematode was prepared in 1,000 µl of buffer, which allowed numerous replications and long-term storage in the freezer for future confirmation and reference. Compared with other real-time-PCR applications [35], [38]–[44], this assay tested more species in *Bursaphelenchus* which include many representative species in nine groups from a total of 13 groups in the genus *Bursaphelenchus*, and more populations of PWN, especially American populations where the nematode originated, and other aphelenchid species. Both PWN-specific and nematode-universal-primer/probe sets using different fluorescent dyes were developed, and the test could be implemented through either simplex- or duplex-real-time PCR. The nematode-universal-primers/probe set for real-time-PCR amplification was included as a nematode endogenous control to detect the presence of nematode-ribosomal-SSU gene, so that a PWN-negative sample can still be evaluated to exclude false negatives due to instrument, pipetting, reagent, and/or reaction failure. In addition, many of the real-time-PCR results were further confirmed by DNA sequencing with GenBank accession numbers (Table 1). This real-time-PCR assay is rapid (<3 h) and therefore ensures a short turnaround time for phytosanitory certification. If a sample of any of these tests is negative for PWN, this study provided a PCR and DNA sequencing approach on ribosomal DNA SSU, LSU D2/D3, ITS and mtCOI genes to help determine the species.
